# Supplementary material for: Mutations in PCYT2 disrupt etherlipid biosynthesis and cause a complex hereditary spastic paraplegia
Source: Brain. 2019 Oct 22;142(11):3382–97. doi: 10.1093/brain/awz291 (PMC6821184; doi:10.1093/brain/awz291)
Supplement: awz291_Supplementary_Data [file awz291_supplementary_data.zip › awz291-Suppl_data/Supplementary_Data4.zip!/Supplementary information 4 - plasma lipidomics.zip!/Plasma lipidomics/Control vs Patient3b/Statistical plots and data/PCA_scores.pdf]

Scores (PCA)

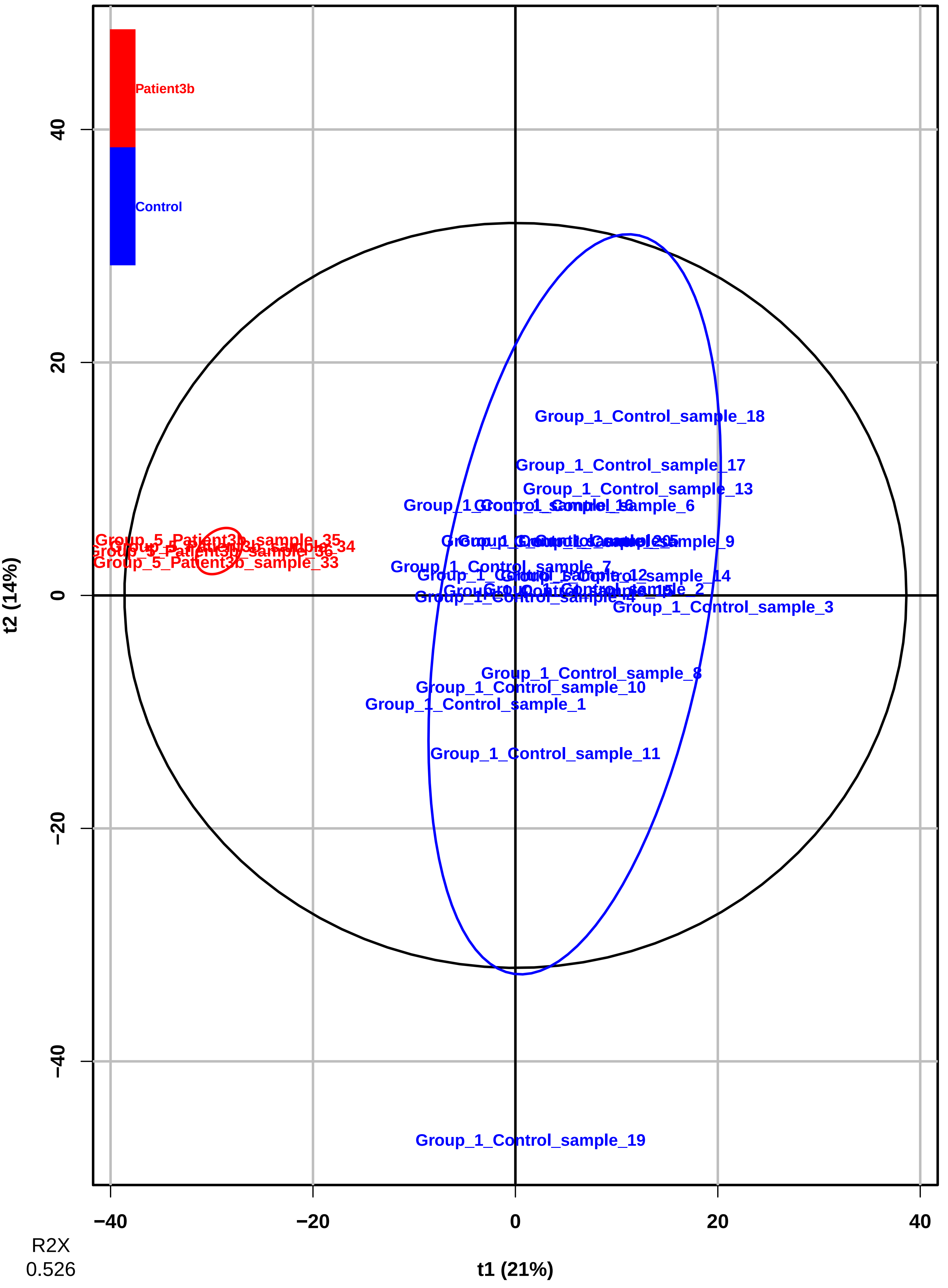
Patient3b
Control
Group_1_Control_sample_18
Group_1_Control_sample_17
Group_1_Control_sample_13
Group_5_Patient3b_sample_35
Group_5_Patient3b_sample_33
Group_1_Control_sample_7
Group_1_Control_sample_3
Group_1_Control_sample_8
Group_1_Control_sample_10
Group_1_Control_sample_1
Group_1_Control_sample_11
Group_1_Control_sample_19
t1 (21%)
t2 (14%)
R2X
0.526
